# Effects of a situational interest-based rotational fitness training program on positive affect in university students: A randomized controlled trial

**DOI:** 10.1097/MD.0000000000050663

**Published:** 2026-09-11

**Authors:** Yin Zhou, Yujia Wu, Qian Su

**Affiliations:** a Capital University of Physical Education and Sports, Beijing, China; b Changzhi University, Changzhi, Shanxi, China; c North University of China, Taiyuan, Shanxi, China.

**Keywords:** EMA, fitness training, positive affect, RCT, situational interest theory, university students

## Abstract

**Background:**

To evaluate whether a 12-week situational interest-based rotational fitness program improved positive emotional states in university students, to compare module-specific effects, and to examine whether the intervention moderated the heart rate–emotion association.

**Methods:**

In this 2-arm randomized controlled trial, 560 freshmen from 10 Chinese universities were assigned to an experimental (n = 280) or control group (n = 280). The experimental group completed weekly 90-minute sessions covering strength, endurance, speed, and agility modules; controls received regular physical education. Positive emotional states were assessed using the positive well-being subscale of the Subjective Exercise Experiences Scale through ecological momentary assessment at 3 points per session, yielding over 14,000 observations. Linear mixed-effects models tested group, time, group × time, heart rate, and heart rate × group effects. Assessors and analysts were blinded. Ethical approval for the trial was granted by the Institutional Review Board of Capital University of Physical Education and Sports (2025A055).

**Results:**

Improvement was greater in the experimental group than in controls (EMM change: 1.843 vs 0.505; difference = 1.338, 95% confidence interval [CI]: 1.275–1.400, *P* < .001). At week 12, the raw between-group difference was 1.85 (*d* = 2.23), and all group × week interactions from weeks 2 to 12 were significant. Heart rate positively predicted emotional states in controls (*B* = 0.005, 95% CI: 0.003–0.007), but this association was attenuated in the experimental group (interaction *B* = −0.008, 95% CI: −0.011 to −0.005). Strength and agility showed significant improvements, while endurance produced consistently higher emotional states across assessment points. Heart rate moderation was significant for strength and endurance, but not speed or agility.

**Conclusion:**

The rotational program improved positive emotional states more than regular physical education, although effects varied by module. Strength showed the most robust benefit. The program also weakened, but did not eliminate, the heart rate–emotion association during more demanding activities.

## 1. Introduction

University students face a substantial burden of mental health issues, particularly depression and anxiety. A recent systematic review and meta-analysis of 64 studies covering 1,00,187 college students reported pooled prevalence rates of 33.6% for depressive symptoms and 39.0% for anxiety symptoms worldwide.^[1]^ In China, a meta-analysis of 113 studies involving 1,85,787 university students estimated the overall prevalence of depression at 28.4%.^[2]^ While recent province-specific epidemiological evidence remains limited, an earlier survey of 1221 students from 3 universities in Shanxi Province showed that students’ SCL-90 scores were significantly higher than national youth norms. This finding suggests that mental health among university students is also a concern in this regional context.^[3]^ Physical activity is widely recognized as beneficial for reducing depression and anxiety and promoting general mental well-being.^[4]^ However, college students often engage in insufficient physical activity, as previous evidence indicates that approximately 40% to 50% of college students are physically inactive.^[5]^ One possible reason is that traditional fitness programs tend to prioritize physiological outcomes, such as strength, endurance, and performance, while paying less attention to students’ emotional experiences during exercise. This mismatch matters because affective responses during and after exercise are highly relevant to future physical activity motivation and behavior.^[6,7]^ Therefore, improving positive affect (PA) in university physical education may become a key strategy to support sustained exercise participation.

Situational interest theory offers a useful framework for designing exercise experiences with higher participation and confidence. According to the 4-phase model of interest development, situational interest can be triggered by features such as novelty, exploration, challenge, immediate feedback, and social interaction.^[8,9]^ In physical education settings, situational interest is closely tied to learning task design. The reason is that students’ interest may be shaped by the task novelty, challenge, exploration, attention demand, and immediate enjoyment.^[10]^ These elements can be applied differently across fitness modules. For example, strength training may foster interest through varied equipment, partner-based tasks, and immediate performance feedback; endurance training may require segmented goals, peer support, and progressive challenge to reduce monotony and fatigue; speed training may use short, high-intensity tasks and rhythm variations to create challenge; and agility training may use game-based tasks, unpredictable movement patterns, and group interaction to increase exploration and enjoyment. Therefore, different fitness modules may not only impose different physiological demands but may also trigger PA through different situational interest mechanisms. However, it remains unclear whether fitness components such as strength, endurance, speed, and agility can respond similarly to a situational interest-based design.

The affective effects of exercise may also depend on the interplay between psychological design and physiological responses. Dual-mode theory suggests that affective responses during exercise are shaped by both cognitive factors and interoceptive reflexes from the body.^[11,12]^ Heart rate can therefore serve as an indicator of physiological load, while PA reflects students’ subjective emotional experience during exercise. A situational interest-based design may alter how students meet physiological demands, but it remains unclear whether the association between heart rate and PA differs between interest-based and traditional training conditions. A closer examination of this relationship can help clarify whether psychologically engaging exercise tasks are associated with an altered or attenuated connection between physiological load and PA. Previous exercise-affect studies often rely on retrospective self-reports, which are vulnerable to recall bias and may fail to capture within-session changes in affect.^[13-15]^ Ecological momentary assessment (EMA) is particularly valuable in physical activity research because it enables repeated, real-time assessment of affective and contextual processes close to their occurrence.^[16]^ In the present study, EMA was administered 3 times during each 90-minute training session. The first 30-minute assessment was designed to capture early affective responses after students entered the training state; the second 60-minute assessment aimed to capture affective responses when physiological load and fatigue were likely to have accumulated; and the final assessment at the end of the session captured immediate postexercise emotional experiences, such as enjoyment, accomplishment, or fatigue-related affect. This 3-point design enabled us to examine dynamic changes in PA within each exercise session.

This randomized controlled trial (RCT) was conducted across 10 universities in Shanxi Province, China. It examined the effects of a 12-week situational interest-based rotational fitness program on PA among university students. The program included strength, endurance, speed, and agility modules. The control group received regular physical education. Three hypotheses were tested: students in the experimental group would show greater improvements in PA than those in the control group; intervention effects would differ across fitness modules due to variation in task characteristics, physiological demands, and situational interest triggers; group condition would moderate the association between heart rate and PA. Specifically, this relationship would be altered or attenuated in the situational interest-based condition. By combining a rotational module design with intensive EMA, this study provides multi-center evidence from universities in Shanxi Province. It also offers a more fine-grained understanding of how theory-based fitness training may shape PA in university physical education.

## 2. Materials and methods

### 2.1. Ethics approval and consent to participate

The study adhered to the Declaration of Helsinki and received approval from the Institutional Review Board of Capital University of Physical Education and Sports (Approval No. 2025A055). All participants provided written informed consent before enrollment. This study was designed and conducted as a prospective RCT. It commenced on September 1, 2025, and was completed on February 3, 2026. The trial record was registered at ClinicalTrials.gov on May 29, 2026 (Registration No. NCT07635927). The present manuscript reports a sub-study of the registered parent trial, focusing on PA and the relationship between heart rate and PA during the 12-week situational interest-based rotational fitness program.

Since EMA data were collected via a mobile application, all app-based data were treated as confidential research data. Participants who completed assessments could use study identification codes rather than personal names. Collected data were de-identified prior to analysis, stored in password-protected research files, and only accessible to authorized members of the research team. No personally identifiable information was disclosed to individuals outside the research team. Information about app-based data collection, confidentiality, and voluntary participation was also included in the informed consent process.

### 2.2. Study design

This study used a 2-arm, parallel-group RCT designed to evaluate the effects of a 12-week situational interest-based rotational fitness program on PA among university students. Allocation was made in equal proportions, with participants assigned to the experimental group and the control group at a 1:1 ratio. The trial was conducted across 10 universities in Shanxi Province, China. The study protocol complied with the Consolidated Standards of Reporting Trials (CONSORT) 2010 guidelines. No major changes were made to the study design, eligibility criteria, intervention procedures, or outcome assessments after its commencement.

### 2.3. Participants

Participants were recruited from 10 universities in Shanxi Province, China, between September and December 2025. A total of 560 first-year undergraduate students were screened and enrolled. Inclusion criteria included: first-year undergraduates; aged 18 to 19 years; no contraindications to physical exercise, as assessed by the Physical Activity Readiness Questionnaire; willingness to participate in the study. Exclusion criteria included: prior participation in structured fitness training within the past 6 months; musculoskeletal injuries that precluded participation in physical exercise; diagnosed mental health conditions; incomplete baseline assessments. The restriction to first-year students aged 18 to 19 years aimed to reduce age- and grade-related heterogeneity. Also, this restriction focused on students during the early transition to university, a period when physical activity habits and emotional experiences in physical education are still in the process of formation. Students receiving prior structured fitness training were excluded to minimize the influence of previous training experience, baseline fitness familiarity, and the novelty of the intervention content. Otherwise, these factors could confound the situational interest-based design. Randomization placed 280 participants in the experimental group and 280 in the control group, preserving the planned 1:1 allocation ratio. The flow diagram provides further details.

### 2.4. Sample size calculation

We estimated the sample size with G*Power 3.1 (Heinrich Heine University, Düsseldorf, Germany). The initial power estimation was based on the primary between-group comparison of change in PA using an independent 2-group comparison. The calculation specified Cohen’s *d* = 0.40 (a small-to-moderate effect size), a 2-tailed α = 0.05, and statistical power of 0.80, producing an estimate of approximately 99 participants in each group. This figure was rounded up to 100 participants per group. Accounting for an anticipated 20% attrition rate, the minimum adjusted sample size was 125 participants per group, or 250 participants in total.

The present study was conducted across 10 universities, involving repeated EMA measurements over 12 weeks, module-specific analyses, and planned heart rate moderation analyses. For these reasons, the recruitment target was further increased, moving beyond the minimum attrition-adjusted sample size. To ensure balanced recruitment across participating universities and to maintain adequate analyzable EMA data, the final recruitment target was set at 280 participants per group. A total of 560 participants were therefore recruited and randomly allocated at a 1:1 ratio. No interim analyses or stopping guidelines were planned (see Table 1).

**Table 1:** Basic demographic characteristics of participants.

Group (n)	Gender (n)	Age (yr)	Grade
Control group (280)	Male (140), Female (140)	Male: 19 ± 0.5, Female: 19 ± 0.5	Freshman
Experimental group (280)	Male (140), Female (140)	Male: 19 ± 0.5, Female: 19 ± 0.5	Freshman

### 2.5. Randomization and allocation concealment

Within each participating university, participants were randomized in equal proportions to the experimental group and control group using a computer-generated random number sequence produced in SPSS version 29.0 (IBM Corp., Armonk). Randomization was stratified by participating university to preserve allocation balance across study sites. Allocation concealment was maintained by having an independent researcher, separate from participant recruitment and outcome assessment, generate the randomization sequence. Group assignments were stored in opaque, sealed envelopes labeled sequentially and opened only after baseline assessments had been completed. Because of the intervention format, participants and teachers could not be blinded; outcome assessors and data analysts, however, remained blinded to group allocation.

### 2.6. Intervention: situational interest-based rotational fitness program

The experimental group (n = 280) undertook a 12-week rotational fitness program comprising 4 modules, delivered in the following sequence: strength (weeks 1–4), endurance (weeks 5–8), speed (weeks 9–10), and agility (weeks 11–12). Training sessions were held once per week, with each session lasting 90 minutes. Each session included warm-up and flexibility preparation, module-specific training activities, cool-down/stretching, and EMA assessments at the scheduled time points. A minimum attendance rate of 80% was required; participants who missed more than 20% of sessions (i.e., more than 2 sessions) were classified non-adherent but retained in the intention-to-treat (ITT) analysis. Attendance was documented by instructors through weekly session logs, while EMA submission records and heart-rate monitoring records were used as supplementary checks of participation.

All modules were designed based on the Situational Interest Theory,^[8-10]^ incorporating 5 triggering elements: novelty, exploration, challenge, immediate feedback, and social interaction. To ensure intervention consistency across the 10 universities, instructors followed a standardized teaching syllabus and session plan. Before implementation, all instructors received training on the intervention rationale, module activities, safety procedures, heart-rate monitoring, EMA administration, and task difficulty adjustment. During the intervention, instructors completed session logs to record attendance, delivery of planned activities, intensity control, and protocol deviations. Specifically, novelty was introduced through varied equipment and rotating modules; exploration through open-ended movement tasks and creative demonstrations; challenge through progressive task difficulty; immediate feedback through heart-rate monitoring, app prompts, and verbal feedback from instructors; and social interaction through partner-based tasks, group competitions, and small-group activities. Task difficulty was adjusted according to heart-rate responses, perceived exertion, movement quality, and signs of fatigue by modifying resistance, repetitions, sets, movement complexity, sprint distance, or rest intervals. Exercise intensity was monitored via Polar H10 heart-rate monitors (Polar Electro, Finland) to help participants maintain exercise intensity within the target zone of 60% to 80% of individual maximum heart rate during each session. Detailed content of all 4 modules is presented in Table 2.

**Table 2:** Syllabus of physical fitness training course.

Module	Week	Exercise type	Teaching content	Load requirement	Organization format
Theoretical module	Week 1		1. Fitness and health promotion 2. Principles, rules and methods of physical fitness training		Online
	Week 7		3. Physical fitness and nutrition 4. Guidance on fitness methods and techniques		Online
	Week 15		5. Fitness guidance for different environments and seasons 6. Formulation and implementation of fitness plans		Online
Practical module (strength training)	Week 1	Strength training 1	Bodyweight upper body: lucky cat raise, bent-over w stretch, bent-over row Bodyweight core: glute bridge, lateral trunk flexion, supine double knee hug Bodyweight lower body: goblet squat, split squat, side squat Game: partner seat balance	6–8 repetitions per movement, 3–6 sets, 1 min rest between sets	Offline
		Strength training 2	Bodyweight upper body: arnold press, pushup with rotation, bear crawl Bodyweight core: supine scissor legs, kneeling elbow to knee touch, single leg high knees Bodyweight lower body: hip rotation in place, lateral shuffle jump, lateral step jump Game: partner V-sit lean	6–8 repetitions per movement, 3–6 sets, 1 min rest between sets	Offline
	Week 2	Strength training 3	Resistance upper body: chest press, bow and arrow pull, bicep curl Bodyweight core: partial crunch, plank, side plank Bodyweight Lower body: single leg squat, overhead squat, reverse crossover step Group competition: plank	6–8 repetitions per movement, 3–6 sets, 1 min rest between sets	Offline
		Strength training 4	Resistance upper body: lateral raise, reverse fly, overhead press Bodyweight core: glute bridge, skater lunge, V-up with rotation Bodyweight lower body: tuck jump, scissor jump, lunge jump Group competition: unity is strength (tuck jump competition)	6–8 repetitions per movement, 3–6 sets, 1 min rest between sets	Offline
	Week 3	Strength training 5	Resistance upper body: bicep curl, single arm overhead press, bilateral diagonal overhead press Bodyweight core: single leg glute bridge with leg lift, supine bicycle crunch, 3-point plank Bodyweight lower body: lunge with rotation, half squat lateral shuffle, half squat lateral crossover shuffle Group discussion: what daily movements involve the biceps brachii?	6–8 repetitions per movement, 3–6 sets, 1 min rest between sets	Offline
		Strength training 6	Resistance upper body: bilateral diagonal forward overhead press, bilateral rotational lateral chest press, bilateral vertical overhead press Bodyweight core: plank with alternating leg tucks, plank with contralateral leg adduction, side plank with arm abduction Bodyweight lower body: front to back jump, snap kick, shuttlecock jump Group discussion: what is the significance of strength training?	6–8 repetitions per movement, 3–6 sets, 1 min rest between sets	Offline
	Week 4	Strength training 7	Micro-exercise production and demonstration: work in pairs to produce a short video based on the learned content Individual demonstration: choose 1–2 movements voluntarily	None	Offline
		Strength training 8	Group competition: choreography demonstration, work in groups of 4–6, the team is required to fully demonstrate the movement combination of strength training with music, with each group lasting no 13 minutes	None	Offline
Practical module (endurance training)	Week 5	Endurance training 1	Aerobic endurance: aerobic walk exercise 1	Aerobic walk exercise: 30 min without rest	Offline
		Endurance training 2	Aerobic endurance: aerobic walk exercise 2	Aerobic walk exercise: 30 min without rest	Offline
	Week 6	Endurance training 3	Aerobic endurance: aerobic walk exercise 3 Scenario micro-exercise: combine the learned content with life scenarios, complete a 2-minute group demonstration in groups of 3	Aerobic walk exercise: 30 min without rest	Offline
		Endurance training 4	Aerobic endurance (level 1 figure rope skipping): alternating side strike, 2-foot Jump, 2-foot lateral jump, jumping jack team game: group rope skipping	Figure rope skipping exercise: 30 min without rest	Offline
	Week 7	Endurance training 5	Aerobic Endurance (Level 1 Figure Rope Skipping): Lunge Jump, Alternating 2-foot Jump, Heel Tap Jump, Cross Jump Individual Demonstration: 4 volunteers choose their familiar rope skipping movements to demonstrate with music	Figure rope skipping exercise: 30 min without rest	Offline
		Endurance training 6	Anaerobic Endurance: HIIT Training 1: Squat, Jumping Jack, High Knees, Mountain Climber, Jog in Place, Burpee Individual Demonstration: 4 volunteers choose their familiar movements to demonstrate	Group circuit training: hold each movement for 20–25 s, repeat 3–5 sets	Offline
	Week 8	Endurance training 7	Anaerobic Endurance: HIIT Training 2: Squat, Jumping Jack, High Knees, Mountain Climber, Jog in Place, Burpee Group Competition: Compete in groups of 3–5	Group circuit training: hold each movement for 20–25 s, repeat 3–5 sets	Offline
		Strength-endurance training 8	Scenario Micro-exercise: Combine the endurance application scenarios in daily life, create freely, and complete a team sports melodrama performance	None	Offline
Practical module (speed training)	Week 9	Speed training 1	Movement Speed Training: Multi-person Creative Agility Ladder Drill Game: Partner Synchronous Lateral Movement	8–12 repetitions per set, 4–6 sets, 2–3 min rest between sets	Offline
		Speed training 2	Displacement Speed Training: Single Leg Hop to Acceleration Sprint. Perform 10–15 meters of single leg hop first, then switch to 20–30 meters of acceleration sprint after hearing the signal. It is required to switch after completing the exercise once with the left and right feet respectively, and the acceleration sprint should reach the maximum speed. Group Discussion: Creativity of Running, each group needs to put forward new ideas	2–4 repetitions per group, repeat 2–3 sets, 3–5 min rest between sets	Offline
	Week 10	Speed training 3	Displacement Speed Training: Handicap Chase Run. Work in groups of 2–3, keep distance according to speed level, with the faster runner in front. After hearing the signal, start with a standing start and sprint at full speed. The latter chases the former, and the former tries not to be caught by the latter. Run 30 meters and 60 meters. Game: Partner Imitation Run	2–3 repetitions per group, repeat 2–3 sets, 3–5 min rest between sets	Offline
		Speed training 4	Reaction Speed Training: Direction Change Start. Stand with the back facing, quickly turn 180 degrees into a half-squat start after hearing the signal, then sprint 20–30 meters. It is required that the turning movement is rapid, and the start and acceleration sprint speed is fast. Talent Show: Imitate the running posture of an animal and complete a 20-meter run	2–3 repetitions per group, repeat 2–3 sets, 3–5 min rest between sets	Offline
Practical module (agility training)	Week 11	Agility training 1	Agility: Procedural Agility Ladder Drill Group Discussion: Creative Design of Agility Training Methods	2–4 sets, 2–3 min rest between sets	Offline
		Agility training 2	Agility: Randomized Cone Drill Group Competition: Happy Dance, freely design movements, and complete the competition in pairs	2–4 sets, 2–3 min rest between sets	Offline
	Week 12	Agility training 3	Agility: Randomized Mini Hurdle Drill Scenario Micro-exercise: Back and Forth (Shuttle Relay)	2–4 sets, 2–3 min rest between sets	Offline
		Agility training 4	Agility: Creative Demonstration (Combine the learned movements, set your own props, and complete 4*8 beats of movements) Sports Testimonial Recording: 2–3 minutes recording per person	2–4 sets, 2–3 min rest between sets	Offline
Practical module (flexibility training)	Week 1–week 12	Flexibility training 1	Yoga: Prayer Pose, High Lunge Twist, Chair Twist, Eagle Pose, Arm Extension Pose, Warrior I Pose, Extended Side Angle Pose, Windblown Tree Pose	Stretching after warm-up at the beginning of the course, practice 2 movements per class, complete the learning in 4 classes, and practice the complete 8 movements with music from week 3	Offline
		Flexibility training 2	Baduanjin (Eight Section Brocade): Holding Up the Heavens to Regulate the Triple Energizer, Drawing the Bow to Shoot the Eagle on Both Sides, Single Arm Raising to Regulate the Spleen and Stomach, Looking Backward to Heal the 5 Strains and 7 Injuries, Shaking the Head and Wagging the Tail to Extinguish the Heart Fire, Climbing the Feet with Both Hands to Strengthen the Kidney and Waist, Clenching the Fists and Glowering to Increase Strength, 7 Bumps Behind to Eliminate All Diseases	Stretching at the end of the course, practice 2 movements per class, and practice the complete 8 movements with music after 4 classes	Offline

### 2.7. Control group

Participants in the control group (n = 280) attended regular physical education courses arranged by their universities. These classes included traditional fitness activities (e.g., running, calisthenics, basic ball games) conducted once per week for 90 minutes. Unlike the experimental program, the control courses neither followed a standardized situational interest-based rotational module design nor systematically incorporated the 5 situational interest elements: rotating module-based novelty, open-ended exploration tasks, individualized challenge progression, app-based immediate feedback, or structured partner/group interaction. All participants were instructed to avoid additional structured fitness training outside scheduled classes throughout the intervention period, with adherence tracked via weekly self-report logs. Additional exercise participation was monitored using weekly self-report logs and was checked by the staff during the study period.

### 2.8. Outcome measures

#### 2.8.1. Positive affect

The primary outcome was PA, which was assessed using the Subjective Exercise Experiences Scale (SEES).^[17]^ The SEES uses a 7-point Likert scale ranging from 1 (not at all) to 7 (very much). It comprises 3 subscales: positive well-being, psychological distress, and fatigue. The positive well-being subscale (4 items) was used to measure PA, with higher scores representing greater PA. The SEES has shown satisfactory reliability and validity in previous studies (Cronbach’s α = 0.85–0.92). Before formal data collection, the app-based SEES questionnaire was piloted in the study population. It showed acceptable reliability and validity with Cronbach’s α = 0.817 and a validity coefficient of 0.902.

#### 2.8.2. EMA protocol

EMA was used to capture real-time emotional experiences during exercise. During each 90-minute training session, participants completed the SEES positive well-being subscale at 3 time points: T1 (30 minutes after session start), T2 (60 minutes after session start), and T3 (immediately at session end). Assessments were delivered via a dedicated mobile app installed on participants’ smartphones. This app was programmed to send assessment prompts at preset time points. Each assessment took approximately 2 to 3 minutes to complete. Participants were required to submit responses within 10 minutes after receiving the prompt. To reduce participant burden and potential reactivity to repeated prompting, only the brief positive well-being subscale was administered at each EMA time point. Prompts were scheduled at fixed time points to avoid unnecessary interruptions. Participants were informed that there were no right or wrong answers and that their responses would not affect their course evaluation or group assignment.

#### 2.8.3. Heart rate measurement

Heart rate was measured using Polar H10 wearable devices at the same 3 time points as the EMA assessments. Heart-rate values were matched to the EMA time points and used as time-varying physiological indicators in the mixed-effects models.

### 2.9. Data collection procedures

Baseline data (demographic characteristics, baseline PA, and resting heart rate) were collected prior to randomization. All baseline assessments were completed by trained research staff using standardized procedures before group allocation. During the intervention, EMA assessments and heart rate measurements were conducted at each training session. At each 90-minute session, PA was assessed via the mobile app at T1, T2, and T3, and heart-rate data were recorded at the corresponding time points using Polar H10 devices. App submission timestamps and heart-rate records were reviewed after each session to confirm whether EMA responses and physiological measurements were completed within the required assessment window and could be matched by participant, week, and time point. Post-intervention data were collected upon completion of the 12-week program.

Data quality checks were completed before analysis. EMA records were coded as missing if participants failed to submit a response within the 10-minute response window, submitted an incomplete response, or did not attend the corresponding training session. Heart-rate records were screened for physiologically implausible values before analysis. Values beyond the reasonable exercise range, such as extremely high values exceeding the expected physiological range for university students during supervised exercise, were treated as invalid. Invalid heart-rate records were coded as missing and were not used in the heart-rate covariate or heart rate × group interaction analyses. Missing EMA responses, late submissions, incomplete responses, and invalid heart-rate records were documented by group, week, and assessment time point.

### 2.10. Statistical analysis

Data analyses were performed with SPSS version 29.0 (IBM Corp., Armonk) and R version 4.0.2. Linear mixed-effects models (LMMs) were fitted in R using the lme4 and lmerTest packages, and estimated marginal means (EMM) were calculated using the emmeans package. Continuous variables were compared between groups using independent-samples *t*-tests, whereas categorical variables were examined using chi-square tests to evaluate baseline comparability between the experimental and control groups. All primary analyses followed the ITT principle, with participants analyzed according to their original group allocation.

The primary analysis employed LMMs to examine changes in PA across the 12-week intervention. The dependent variable was change in PA from baseline. Group, week, and the group × week interaction were entered as fixed effects with week treated as a categorical variable. A participant-specific random intercept captured the dependence created by repeated observations within the same individual. Also, heart rate was mean-centered and entered as a time-varying covariate. The heart rate × group interaction was included to examine whether the association between heart rate and PA differed between the experimental and control groups.

Given that all universities involved were ordinary undergraduate institutions in Shanxi Province, and that identical intervention syllabus, instructor training procedures, EMA protocol, and heart-rate monitoring procedures were used across sites, university was not modeled as a separate clustering level in the primary analysis. Instead, site-related variation was minimized through standardized intervention delivery and data collection procedures.

Module-specific analyses were conducted using separate LMMs for the strength, endurance, speed, and agility modules. To align with the module transition structure, module-specific change scores were calculated relative to the assessment immediately preceding each module: week 1 for the strength module; week 4 for the endurance module; week 8 for the speed module; and week 10 for the agility module. Group, week, group × week interaction, centered heart rate, and heart rate × group interaction were included as fixed effects in each specific model with participant included as a random intercept. Significant interaction effects were further explored using simple-effects or post hoc comparisons where appropriate.

EMMs were calculated for group comparisons with heart rate controlled in the model. Model estimates were reported with standard error, test statistics, *P* values, and 95% confidence intervals (CIs). Where appropriate, standardized effect sizes were calculated to support the interpretation of the practical magnitude of group differences. A 2-tailed α = 0.05 was adopted for all analyses.

Missing EMA data were handled using restricted maximum likelihood (REML) estimation within the mixed-model framework, which enables all available observations to contribute to parameter estimation under the missing-at-random (MAR) assumption.^[18,19]^ Missing-data patterns were examined by group, week, module, and EMA assessment time point. In order to evaluate the plausibility of the MAR assumption, baseline characteristics were compared between participants with higher and lower EMA completion rates. The robustness of the primary results was additionally evaluated through complete-case sensitivity analyses.

## 3. Results

### 3.1. Participant flow and baseline characteristics

A total of 560 participants were randomly allocated to the 2 groups (280 per group). All these participants were retained in the ITT analyses, and no participant was excluded from the primary analysis after randomization. The overall EMA completion rate was 71.69%, and the pattern of missing data was consistent with the MAR assumption, supporting the appropriateness of REML-based mixed-model estimation. Randomization was successful as evidenced by the baseline characteristics: the groups were well matched on sex distribution (50.00% male, 50.00% female in each group; χ^2^ = 0.000, *P* = 1.000), resting heart rate (experimental: 72.40 ± 0.93 bpm; control: 72.30 ± 0.88 bpm; *t* = 1.353, *P* = .177), and baseline PA (experimental: 3.72 ± 0.23; control: 3.73 ± 0.16; *t* = −0.683, *P* = .495), laying a solid foundation for subsequent comparisons (see Table 3).

**Table 3:** Baseline characteristics of the 2 groups.

Indicator	Experimental group	Control group	Statistical value	*P* value
Sample size, n	280	280	–	–
Male, n (%)	140 (50.00)	140 (50.00)	χ^2^ = 0.000	1.000
Female, n (%)	140 (50.00)	140 (50.00)	–	–
Baseline heart rate	72.40 ± 0.93	72.30 ± 0.88	*t* = 1.353	.177
Baseline positive affect (PA)	3.72 ± 0.23	3.73 ± 0.16	*t* = −0.683	.495

Across the 12-week intervention, the overall EMA completion rate was 71.69%. EMA completion varied across weeks and modules. In the strength module, valid observations increased from week 1 to week 3 and remained relatively high in week 4. During the endurance module, completion was stable, while a lower number of valid observations was observed in week 7. Completion remained relatively stable during the speed module but declined substantially during the agility module, especially in weeks 11 and 12. These findings indicate that missing EMA data were not evenly distributed across the intervention period and were more pronounced in the later weeks.

To further examine the missing-data mechanism, participants with higher EMA completion rates (≥75%) were compared with those with lower completion rates (<75%) on baseline characteristics. The higher- and lower-completion groups did not differ significantly in sex distribution (76/66 vs 72/66 for male/female; χ^2^ = 0.08, *P* = .781), initial exercise-session heart rate (98.4 ± 17.2 bpm vs 99.1 ± 18.0 bpm; *t* = −0.33, *P* = .742), or baseline PA (4.12 ± 0.68 vs 4.08 ± 0.72; t = 0.47, *P* = .637). These findings provided empirical support for the plausibility of the MAR assumption and the use of REML-based mixed-effects models.

Heart-rate data were also screened prior to analysis. Twenty heart-rate records were identified as physiologically implausible values, accounting for approximately 0.1% of all heart-rate records. These values were coded as invalid and treated as missing in analyses involving heart rate as a time-varying covariate or the heart rate × group interaction.

To test robustness across data-handling approaches, we repeated the analysis using complete cases and compared it with the analysis based on all available data. Both approaches produced a consistent intervention pattern. At week 12, the between-group difference in PA was still 1.85 points, while the standardized effect remained large (Cohen’s *d* = 2.23, 95% CI: [1.88, 2.58]). This consistency indicates that the primary finding was robust when the analysis was restricted to participants with available week 12 data.

Model diagnostic indices were also reported to enhance the transparency of the mixed-effects models. For the overall 12-week model and the 4 module-specific models, participant-level intraclass correlation coefficient, Akaike information criterion, Bayesian information criterion, random-intercept variance, residual variance, and total variance are summarized in Table 4.

**Table 4:** Model diagnostic indices for the overall and module-specific linear mixed models.

Model	ICC	AIC	BIC	Random intercept variance	Residual variance	Total variance
Overall 12-week model	0.079	11,432.219	11,613.453	0.051	0.594	0.645
Strength module	0.119	4307.830	4374.053	0.072	0.537	0.609
Endurance module	0.513	4711.440	4776.573	0.648	0.615	1.263
Speed module	0.518	2443.460	2481.403	0.612	0.569	1.181
Agility module	0.476	1066.414	1097.547	0.566	0.623	1.189

AIC = Akaike information criterion, BIC = Bayesian information criterion, ICC = intraclass correlation coefficient.

### 3.2. Overall intervention effect on positive affect

**Figure 1.**
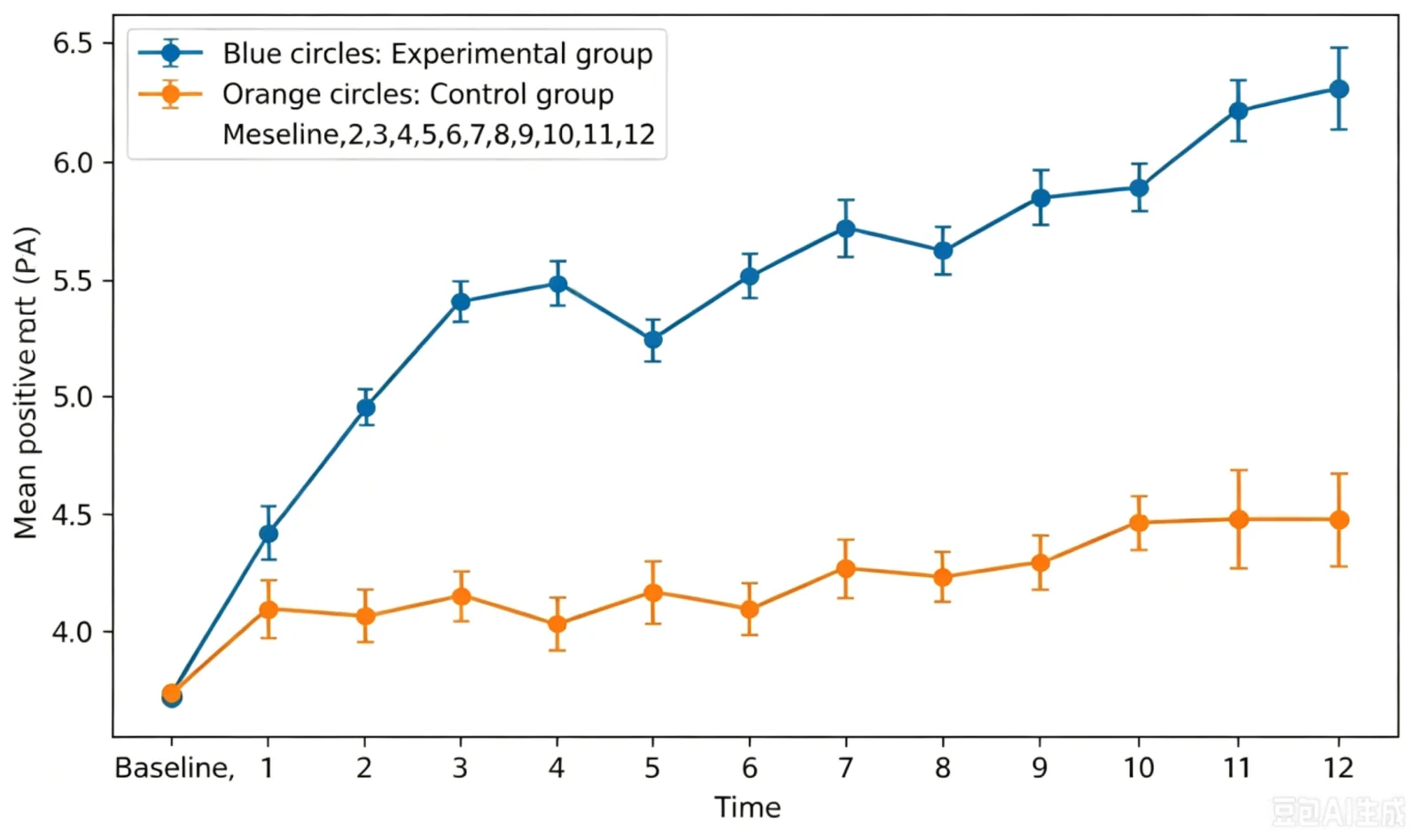
Weekly trajectory of positive affect (PA) in the experimental and control groups across the 12-week intervention period. Error bars indicate variability around the weekly mean. PA = positive affect.

Across the 12 weeks, PA in the experimental group rose steadily, from a mean of 4.41 (standard deviation [SD] = 0.69) at week 1 to 6.31 (SD = 0.79) at week 12. The control group changed only modestly, increasing from 4.10 (SD = 0.80) to 4.46 (SD = 0.87) over the same period. weekly scores favored the experimental group throughout, and the separation between groups became more pronounced after week 3 (see Fig. 1). By week 12, the unadjusted between-group difference in PA was 1.85 points (Cohen’s *d* = 2.23), indicating a large effect in practical as well as statistical terms.

The LMM based on change from baseline supported the descriptive pattern. The group effect was significant (*B* = 0.233, 95% CI: 0.057–0.409, *P* = .010), indicating greater baseline-referenced improvement in the experimental group than in the control group from the week 1 reference point. Positive group × week coefficients were observed at every assessment from week 2 through week 12 (all *P* < .001; coefficients ranging from *B* = 0.557 at week 2 to *B* = 1.623 at week 12), showing that the difference between conditions increased over time. Among controls, heart rate was positively associated with change in PA (*B* = 0.005, 95% CI: 0.003–0.007, *P* < .001). The negative heart rate × group coefficient was also significant (*B* = −0.008, 95% CI: −0.011 to −0.005, *P* < .001), indicating a weaker heart rate–PA association in the experimental condition. Accordingly, this result was interpreted as attenuation of the association rather than complete decoupling. With heart rate held constant, the EMM change estimates were 0.505 for the control group and 1.843 for the experimental group, a between-group difference of 1.338 (95% CI: 1.275–1.400, *P* < .001; see Tables 5 and 6).

**Table 5:** Descriptive statistics of weekly positive affect (PA) Over 12 weeks.

Week	Experimental group, n	Experimental group PA (mean ± SD)	Control group, n	Control group PA (mean ± SD)
1	160	4.41 ± 0.69	165	4.10 ± 0.80
2	255	4.95 ± 0.57	203	4.07 ± 0.82
3	280	5.42 ± 0.70	277	4.15 ± 0.83
4	255	255	247	4.04 ± 0.89
5	240	5.25 ± 0.70	210	4.16 ± 0.95
6	190	5.52 ± 0.64	232	4.10 ± 0.84
7	123	5.72 ± 0.65	247	4.26 ± 0.93
8	224	5.63 ± 0.77	256	4.24 ± 0.82
9	196	5.85 ± 0.79	204	4.29 ± 0.80
10	219	5.90 ± 0.73	237	4.44 ± 0.85
11	120	6.22 ± 0.68	74	4.46 ± 0.89
12	85	6.31 ± 0.79	83	4.46 ± 0.87

PA = positive affect.

**Table 6:** Results of the 12-week linear mixed-effects model (change from baseline).

Fixed effect	*B*	SE	*z*	*P* value	95% CI
Intercept (control group, week 1 change from baseline)	0.443	0.063	7.011	<.001	0.319–0.567
Group (experimental vs control, week 1 difference in change from baseline)	0.233	0.090	2.591	.010	0.057–0.409
Week (week 2 vs week 1, change from baseline)	−0.033	0.081	−0.404	.686	−0.191 to 0.126
Week (week 3 vs week 1, change from baseline)	0.018	0.076	0.234	.815	−0.132 to 0.168
Week (week 4 vs week 1, change from baseline)	−0.089	0.078	−1.138	.255	−0.241 to 0.064
Week (week 5 vs week 1, change from baseline)	−0.034	0.082	−0.415	.678	−0.195 to 0.127
Week (week 6 vs week 1, change from baseline)	−0.085	0.080	−1.062	.288	−0.242 to 0.072
Week (week 7 vs week 1, change from baseline)	0.064	0.080	0.800	.424	−0.092 to 0.219
Week (week 8 vs week 1, change from baseline)	0.034	0.424	0.424	.671	−0.121 to 0.189
Week (week 9 vs week 1, change from baseline)	0.057	0.085	0.672	.501	−0.110 to 0.224
Week (week 10 vs week 1, change from baseline)	0.198	0.082	2.406	.016	0.037–0.359
Week (week 11 vs week 1, change from baseline)	0.290	0.111	2.616	.009	0.073–0.507
Week (week 12 vs week 1, change from baseline)	0.330	0.106	3.123	.002	0.123–0.536
Interaction (experimental group × week 2, change from baseline)	0.557	0.113	4.942	<.001	0.336–0.778
Interaction (experimental group × week 3, change from baseline)	0.971	0.108	8.948	<.001	0.758–1.183
Interaction (experimental group × week 4, change from baseline)	1.152	0.110	10.435	<.001	0.936–1.369
Interaction (experimental group × week 5, change from baseline)	0.910	0.115	7.912	<.001	0.684–1.135
Interaction (experimental group × week 6, change from baseline)	1.232	0.116	10.606	<.001	1.004–1.459
Interaction (experimental group × week 7, change from baseline)	1.246	0.123	10.127	<.001	1.004–1.487
Interaction (experimental group × week 8, change from baseline)	1.216	0.114	10.697	<.001	0.993–1.438
Interaction (experimental group × week 9, change from baseline)	1.430	0.120	11.928	<.001	1.195–1.665
Interaction (experimental group × week 10, change from baseline)	1.332	0.117	11.402	<.001	1.103–1.561
Interaction (experimental group × week 11, change from baseline)	1.590	0.146	10.880	<.001	1.303–1.876
Interaction (experimental group × week 12, change from baseline)	1.623	0.149	10.888	<.001	1.331–1.915
Heart rate (centered, time-varying covariate)	0.005	0.001	4.829	<.001	0.003–0.007
Heart rate × group interaction	−0.008	0.002	−5.321	<.001	−0.011 to −0.005

CI = confidence interval.

### 3.3. Module-specific effects

Separate LMMs were fitted for each fitness module, with module-specific baselines. To ensure consistency in the reporting across modules, model-based estimates are presented alongside EMM differences, 95% CIs, and end-of-module standardized between-group effects where appropriate. Model diagnostic indices for all module-specific models are reported in Table 4.

#### 3.3.1. Strength module (weeks 1–4)

At module entry, the experimental group had a significant advantage (*B* = 0.312, 95% CI: 0.143–0.481, *P* < .001). The group × week coefficients were positive at weeks 2, 3, and 4 (*B* = 0.566, 0.975, and 1.158, respectively; all *P* < .001), showing progressively larger intervention-related improvement across the strength module. In the control group, heart rate was positively associated with change in PA (*B* = 0.003, 95% CI: 0.001–0.006, *P* = .014), consistent with modest physiological–emotional coupling under traditional training. The experimental condition significantly weakened this association (heart rate × group: *B* = −0.004, 95% CI: −0.008 to −0.000, *P* = .041). Thus, the heart rate–PA association was weaker in the experimental group, a pattern interpreted as attenuation rather than complete decoupling. The between-group EMM contrast was substantial (1.352 vs 0.366; difference = 0.986, 95% CI: 0.903–1.069, *P* < .001). At week 4, the unadjusted group difference was 1.45 points, representing a large standardized effect (Cohen’s *d* = 1.75; see Table 7).

**Table 7:** Results of linear mixed-effects model for strength module (weeks 1–4; change from baseline).

Fixed effect	*B*	SE	*z*	*P* value	95% CI
Intercept (control group, change from baseline in week 1)	0.389	0.060	6.438	<0.001	0.271–0.508
Group (experimental vs control, difference in change from baseline in week 1)	0.312	0.086	3.615	<0.001	0.143–0.481
Week (week 2 vs week 1, change from baseline)	−0.031	0.077	−0.395	0.693	−0.182 to 0.121
Week (week 3 vs week 1, change from baseline)	0.023	0.073	0.311	0.756	−0.121 to 0.166
Week (week 3 vs week 1, change from baseline)	−0.087	0.074	−1.167	0.243	−0.233 to 0.059
Interaction (experimental group × week 2, change from baseline)	0.566	0.108	5.265	<0.001	0.355–0.777
Interaction (experimental group × week 3, change from baseline)	0.975	0.104	9.382	<0.001	0.771–1.179
Interaction (experimental group × week 4, change from baseline)	1.158	0.106	10.959	<0.001	0.950–1.365
Heart rate (centered, time-varying covariate)	0.003	0.001	2.456	0.014	0.001–0.006
Heart rate × group interaction	−0.004	0.002	−2.042	0.041	−0.008 to −0.000

CI = confidence interval.

#### 3.3.2. Endurance module (weeks 5–8)

Using week 4 as the reference, the week 5 group coefficient was significant and negative (*B* = −0.362, 95% CI: −0.569 to −0.155, *P* < .001). The subsequent group × week coefficients were positive and significant (week 6: *B* = 0.297, *P* = .007; week 7: *B* = 0.349, *P* = .003; week 8: *B* = 0.320, *P* = .002), suggesting recovery across the endurance module. Because the initial coefficient was negative, additional simple-effect analyses were conducted. At every endurance-week assessment, PA remained significantly higher in the experimental group than in the control group: week 5 mean difference = 1.09, *P* < .001; week 6 mean difference = 1.42, *P* < .001; week 7 mean difference = 1.46, *P* < .001; and week 8 mean difference = 1.39, *P* < .001. Within the experimental group, PA increased from week 5 to week 8 (Δ = 0.38, *P* < .001), whereas the corresponding change in the control group was not significant (Δ = 0.08, *P* = .330). In the control group, heart rate was positively related to positive-affect change (*B* = 0.010, 95% CI: 0.006–0.013, *P* < .001). The significant negative heart rate × group coefficient (*B* = −0.019, 95% CI: −0.025 to −0.013, *P* < .001) showed that this association was substantially weaker in the experimental group during endurance training. This moderation result should be treated cautiously; by itself, it does not demonstrate a confirmed decoupling mechanism. The overall EMM contrast for change from week 4 did not reach significance (experimental: 0.062; control: 0.183; difference = −0.121, 95% CI: −0.285–0.044, *P* = .152). At week 8, the unadjusted between-group difference was 1.39 points, equivalent to a large standardized effect (Cohen’s *d* = 1.74; see Table 8).

**Table 8:** Results of linear mixed-effects model for endurance module (weeks 5–8; change from week 4).

Fixed effect	*B*	SE	*z*	*P* value	95% CI
Intercept (control group, change from week 4 in week 5)	0.155	0.075	2.059	0.040	0.007–0.303
Group (experimental vs control, difference in change from week 4 in week 5)	−0.362	0.106	−3.429	<0.001	−0.569 to −0.155
Week (week 6 vs week 5, change from week 4)	−0.032	0.077	−0.415	0.678	−0.182 to 0.118
Week (week 7 vs week 5, change from week 4)	0.087	0.075	1.163	0.245	−0.060 to 0.234
Week (week 8 vs week 5, change from week 4)	0.054	0.075	0.729	0.466	−0.092 to 0.200
Interaction (experimental group × week 6, change from week 4)	0.297	0.109	2.713	0.007	0.082–0.511
Interaction (experimental group × week 7, change from week 4)	0.349	0.118	2.956	0.003	0.118–0.580
Interaction (experimental group × week 8, change from week 4)	0.320	0.105	3.049	0.002	0.114–0.525
Heart rate (centered, time-varying covariate)	0.010	0.002	4.770	<0.001	0.006–0.013
Heart rate × group interaction	−0.019	0.003	−6.427	<0.001	−0.025 to −0.013

CI = confidence interval.

#### 3.3.3. Speed module (weeks 9–10)

No module-specific effect reached statistical significance. The group term (*B* = 0.163, 95% CI: −0.053–0.379, *P* = .138), time term (*B* = 0.131, *P* = .082), and group × time term (*B* = −0.092, *P* = .389) were all nonsignificant. Neither heart rate (*B* = 0.002, *P* = .705) nor the group × heart rate interaction (*B* = −0.006, *P* = .437) was significant. Thus, relative to the week 8 module baseline, the speed module produced no detectable additional change in PA and no evidence of heart rate moderation. The EMM contrast was also nonsignificant (0.249 vs 0.132; difference = 0.117, 95% CI: −0.063–0.297, *P* = .203). Although the unadjusted week 10 group difference remained large (mean difference = 1.46, Cohen’s *d* = 1.84), this value is best treated as a descriptive end-of-module contrast rather than evidence of a significant speed-specific LMM effect (see Table 9).

**Table 9:** Results of linear mixed-effects model for speed module (weeks 9–10; change from week 8).

Fixed effect	*B*	SE	*z*	*P* value	95% CI
Intercept (control group, change from week 8 in week 9)	0.066	0.077	0.857	0.392	−0.085 to 0.217
Group (experimental vs control, difference in change from week 8 in week 9)	0.163	0.110	1.482	0.138	−0.053 to 0.379
Week (week 10 vs week 9, change from week 8)	0.131	0.076	1.737	0.082	−0.017 to 0.280
Interaction (experimental group × week 10, change from week 8)	−0.092	0.107	−0.861	0.389	−0.302 to 0.118
Heart rate (centered, time-varying covariate)	0.002	0.005	0.378	0.705	−0.008 to 0.012
Heart rate × group interaction	−0.006	0.008	−0.778	0.437	−0.022 to 0.010

CI = confidence interval.

#### 3.3.4. Agility module (weeks 11–12)

Evidence of an intervention effect reemerged at the start of this module. At week 11, the group term was positive and significant (*B* = 0.374, 95% CI: 0.054–0.694, *P* = .022). Neither the week 12 effect nor the group × week interaction was significant, suggesting that the initial agility-module advantage persisted without further amplification. Heart-rate terms were nonsignificant in both groups (main effect: *B* = 0.002, *P* = .834; interaction: *B* = 0.002, *P* = .905), providing no evidence of heart rate moderation in this module. The between-group EMM contrast was significant (0.355 vs −0.048; difference = 0.403, 95% CI: 0.135–0.672, *P* = .003). By week 12, the unadjusted group difference was 1.85 points, equivalent to a large standardized effect (Cohen’s *d* = 2.23). Because the agility module lasted only 2 weeks and EMA completion was lower in weeks 11 to 12, this result warrants cautious interpretation (see Table 10).

**Table 10:** Results of linear mixed-effects model for agility module (weeks 11–12; change from week 10).

Fixed effect	*B*	SE	*z*	*P* value	95% CI
Intercept (control group, change from week 10 in week 11)	−0.049	0.128	−0.383	0.702	−0.299 to 0.201
Group (experimental vs control, difference in change from week 10 in week 11)	0.374	0.163	2.292	0.022	0.054 to 0.694
Week (week 12 vs week 11, change from week 10)	0.001	0.144	0.008	0.994	−0.282 to 0.284
Interaction (experimental group × week 12, change from week 10)	0.059	0.189	0.310	0.757	−0.312 to 0.429
Heart rate (centered, time-varying covariate)	0.002	0.011	0.210	0.834	−0.018 to 0.023
Heart rate × group interaction	0.002	0.015	0.119	0.905	−0.027 to 0.030

CI = confidence interval.

## 4. Discussion

This RCT, conducted across 10 universities in Shanxi Province, China, utilized EMA with more than 14,000 real-time observations to evaluate a 12-week situational interest-based rotational fitness program. The experimental condition produced a markedly larger increase in PA than regular physical education, and the magnitude of this difference was substantial both statistically and practically. Rather than merely reflecting general time-related change, the pattern of significant group × week interactions suggested that the affective improvement was more closely associated with the intervention condition. The rotational design may have helped sustain PA by repeatedly introducing novelty, challenge, exploration, feedback, and social interaction, which are central components of situational interest in physical education settings.^[6,20]^

The differential module effects should be considered carefully. The strength module yielded robust between-group differences and a significant heart rate × group interaction. However, this pattern should be interpreted as an attenuation of the heart rate–PA association rather than as evidence of complete physiological–emotional decoupling. Strength training may be particularly well suited to an interest-based design because partner exercises, varied equipment, progressive challenges, and immediate feedback can make the activity more engaging and personally responsive. These features may reduce the extent to which PA depends on physiological exertion alone. That said, the present study did not directly test this psychological pathway. The agility module, which is characterized by game-like, exploratory, and unpredictable tasks, also showed a significant between-group difference in affective change. This finding is consistent with the proposed affective value of playful and exploratory movement tasks. Given the 2-week duration of the agility module and the lower EMA completion in the later weeks, the agility findings warrant cautious interpretation.

Interpretation of the endurance findings is less straightforward. The initial group effect was negative when change was calculated relative to the preceding strength module, which may partly reflect a module-transition effect rather than a true decline in the intervention group’s overall affective advantage. Additional simple-effect analyses showed that the experimental group still reported higher PA than the control group at each endurance-week assessment, and PA increased significantly from week 5 to week 8 in the experimental group. Consistent with dual-mode theory,^[9]^ sustained aerobic exercise may produce stronger interoceptive cues such as fatigue and breathlessness, which can reshape affective responses during higher-demand activity. The endurance module showed the strongest heart rate moderation effect, with a positive heart rate–PA association in the control group and a weaker association in the experimental group. This finding suggests that situational interest-based design may have reduced the affective impact of physiological demand during endurance training. The moderation result alone cannot confirm a physiological-affective decoupling mechanism. This interpretation is also consistent with the hedonic approach to exercise, which highlights the importance of affective responses in shaping exercise experience and future participation.^[14,21]^

The speed module did not show significant module-specific effects. Accordingly, no firm conclusion should be drawn about its transitional role without additional adjacent-module contrasts or counterbalanced module-order designs. The nonsignificant group effect, group × week interaction, heart rate effect, and heart rate × group interaction indicate that the speed module did not produce additional positive-affect change beyond the prior module baseline. Because both the speed and agility modules lasted only 2 weeks, their nonsignificant heart-rate moderation effects may reflect shorter observation windows and reduced statistical power to some extent. However, this possibility remains tentative and should be examined in future studies with longer module durations or balanced module sequences.

Taken together, the module-specific patterns suggest that situational interest-based rotational training may enhance PA across different fitness contents, but the magnitude and form of this effect can vary by module. The findings provide preliminary evidence that interest-based design may be especially beneficial in modules where physiological demand is high or where tasks can be made exploratory, social, and feedback-rich. However, the present results do not directly establish the psychological mechanism. Future research should include validated measures of perceived situational interest, such as the Situational Interest Inventory for physical education, and test whether perceived interest mediates the relationship between module design and affective response.^[20]^

Methodological strengths of this study include the use of EMA, which provided real-time evaluations and reduced reliance on retrospective recall. However, intensive EMA also has limitations. Repeated app-based assessments may increase participant burden, response fatigue, and missing responses, particularly in longer protocols.^[22,23]^ The large sample size and implementation across 10 universities also strengthened the ecological validity of the study within ordinary university physical education settings in Shanxi Province. Nevertheless, because all participating universities were located in a single province and the sample consisted of first-year students aged 18 to 19 years, geographic, developmental, and cultural generalizability remains limited.

Several limitations should be acknowledged. First, the fixed module order may have introduced order effects; future counterbalanced or randomized module-sequence designs are needed to address this. Second, no long-term follow-up was conducted. Future studies should examine whether short-term improvements in PA translate into sustained physical activity, objective adherence, and longer-term mental health or well-being outcomes.^[23,24]^ Third, reliance on self-reported PA may introduce response bias, while the EMA design reduced retrospective recall bias. Future studies could combine EMA with additional behavioral or psychophysiological indicators of affective response. Fourth, direct assessments of perceived situational interest were not collected, leaving the underlying mediating mechanism inferential. Future research should measure perceived interest repeatedly across modules and test formal mediation models linking situational interest, affective response, and exercise adherence.^[20]^ Finally, the shorter duration of the speed and agility modules may have constrained statistical power for detecting module-specific moderation effects. Longer and more balanced module designs are needed to determine whether the observed heart rate–PA patterns generalize across different exercise modalities.

## 5. Conclusion

In conclusion, a 12-week situational interest-based rotational fitness program was associated with greater improvements in PA among first-year university students compared with regular physical education training. Intervention effects varied across fitness modules, with robust affective benefits observed during the strength module and positive but more cautiously interpreted effects during the later agility module. Situational interest-based design may attenuate the association between heart rate and PA in some exercise contexts, especially during higher-demand activities. However, the present findings do not establish a complete physiological–emotional decoupling mechanism. These findings provide preliminary empirical support for incorporating situational interest theory into university physical education reform, with the aim of fostering more positive affective experiences during exercise.

## Acknowledgments

We thank the participating students and university staff from the 10 universities in Shanxi Province for supporting study implementation and data collection.

## Author contributions

**Conceptualization:** Yin Zhou.

**Data curation:** Yujia Wu.

**Formal analysis:** Qian Su.

**Investigation:** Yin Zhou, Yujia Wu, Qian Su.

**Methodology:** Yin Zhou.

**Writing – original draft:** Yin Zhou.

**Writing – review & editing:** Yin Zhou.
